# DNA methylation among firefighters

**DOI:** 10.1371/journal.pone.0214282

**Published:** 2019-03-26

**Authors:** Jin Zhou, Timothy G. Jenkins, Alesia M. Jung, Kyoung Sook Jeong, Jing Zhai, Elizabeth T. Jacobs, Stephanie C. Griffin, Devi Dearmon-Moore, Sally R. Littau, Wayne F. Peate, Nathan A. Ellis, Peter Lance, Yin Chen, Jefferey L. Burgess

**Affiliations:** 1 Department of Epidemiology and Biostatistics, Mel and Enid Zuckerman College of Public Health, University of Arizona, Tucson, Arizona, United States of America; 2 Department of Surgery, University of Utah School of Medicine, Salt Lake City, Utah, United States of America; 3 Department of Occupational and Environmental Medicine, Hallym University Sacred Heart Hospital, Anyang, Republic of Korea; 4 University of Arizona Cancer Center, Tucson, Arizona, United States of America; 5 Department of Community, Environment and Policy, Mel and Enid Zuckerman College of Public Health, University of Arizona, Tucson, Arizona, United States of America; 6 WellAmerica, Tucson, Arizona, United States of America; 7 Department of Pharmacology and Toxicology, College of Pharmacy, University of Arizona, Tucson, Arizona, United States of America; University of Bonn, Institute of Experimental Hematology and Transfusion Medicine, GERMANY

## Abstract

Firefighters are exposed to carcinogens and have elevated cancer rates. We hypothesized that occupational exposures in firefighters would lead to DNA methylation changes associated with activation of cancer pathways and increased cancer risk. To address this hypothesis, we collected peripheral blood samples from 45 incumbent and 41 new recruit non-smoking male firefighters and analyzed the samples for DNA methylation using an Illumina Methylation EPIC 850k chip. Adjusting for age and ethnicity, we performed: 1) genome-wide differential methylation analysis; 2) genome-wide prediction for firefighter status (incumbent or new recruit) and years of service; and 3) Ingenuity Pathway Analysis (IPA). Four CpGs, including three in the *YIPF6*, *MPST*, and *PCED1B* genes, demonstrated above 1.5-fold statistically significant differential methylation after Bonferroni correction. Genome-wide methylation predicted with high accuracy incumbent and new recruit status as well as years of service among incumbent firefighters. Using IPA, the top pathways with more than 5 gene members annotated from differentially methylated probes included Sirtuin signaling pathway, p53 signaling, and 5' AMP-activated protein kinase (AMPK) signaling. These DNA methylation findings suggest potential cellular mechanisms associated with increased cancer risk in firefighters.

## Introduction

Epidemiologic studies of firefighters from multiple countries have demonstrated an elevated rate of cancer incidence and/or mortality for a number of cancer types [1–8]. For example, in a recent study of three large fire departments in the United States, overall cancer incidence and mortality was significantly increased by 9% and 14%, respectively, as compared with the general population, and significant increases in cancer incidence and mortality were noted specifically for cancers of the esophagus, intestine, lung, and kidney [2]. Firefighters are occupationally exposed to multiple products of combustion and other substances containing carcinogens through inhalation and/or skin contamination [9–11], including but not limited to polycyclic aromatic hydrocarbons (PAHs), benzene, per- and polyfluoroalkyl substances (PFAS) and diesel exhaust [11–15]. However, other risk factors such as shift work may also contribute to this elevated cancer risk [16].

Epigenetic modifications are critical steps in carcinogenesis and cancer prevention [17, 18]. We have previously shown that microRNAs are differentially expressed between incumbent and new recruit firefighters [19], but published information on DNA methylation in firefighters to our knowledge has been limited to four genes [20]. DNA methylation refers to the addition of a methyl group to cytosine within 5'-C-phosphate-G-3' (CpG) dinucleotides, which are often concentrated in large clusters called CpG islands. Inactivation of certain tumor-suppressor genes occurs as a consequence of hypermethylation within the promoter regions and numerous studies have demonstrated a broad range of genes silenced by DNA methylation in different cancer types [21–24]. Global hypomethylation, inducing genomic instability, also contributes to cell transformation. Apart from DNA methylation alterations in promoter regions and repetitive DNA sequences, this phenomenon is associated with regulation of expression of noncoding RNAs such as microRNAs that may play a role in tumor suppression. Furthermore, DNA methylation has shown promise in putative translational use in patients and hypermethylated promoters may serve as disease-related biomarkers [25, 26]. Importantly, while every effort is made by previous studies to put identified methylation signatures in context, it should be noted that the landscape of methylation alterations and the associated impact on gene activity is extremely complex. Thus, changes in methylation signatures are not always clearly linked to specific alterations in gene activity.

We hypothesized that compared to new recruits without previous firefighting experience, incumbent firefighters would show differential DNA methylation patterns that had been previously associated with cancer. We analyzed DNA methylation in peripheral blood by microarray and compared the results between new recruits and incumbent firefighters to address this hypothesis.

## Methods

### Subjects

Study protocols were approved by the University of Arizona Institutional Review Board (approval No.1509137073) and all subjects provided written informed consent. The study subjects were selected from a larger group of incumbent firefighters within the Tucson Fire Department (Tucson, Arizona, United States of America) and new recruit firefighters prior to any live-fire exposures or other occupational exposures to fire and smoke. All subjects completed questionnaires regarding their age, body weight, height, working duration as firefighters, and tobacco use.

Initially, blood for methylation analysis was collected from 47 male incumbents and 48 male and one female new recruits. Subjects who either had current smoking exposure or missing smoking information and the sole female recruit were excluded, leaving 86 (45 incumbents and 41 recruits) subjects for methylation data analysis. Body mass index (BMI) (kg/m^2^) was classified as normal (18.5–24.9), overweight (25.0–29.9), and obese (≥ 30) following World Health Organization (WHO) classifications.

### DNA methylation measurement

Blood samples were collected in one 6.0 ml dipotassium ethylenediaminetetraacetic acid (K_2_EDTA) tube (Becton, Dickinson and Company, Franklin Lakes, NJ) for DNA methylation analyses. As an alternative to the ethylenediaminetetraacetic acid (EDTA) tube, eight samples were also collected in cell preparation tubes (CPTs) (Becton, Dickinson and Company, Franklin Lakes, NJ). The EDTA tube was processed within 30 minutes of collection, which consisted of centrifugation at 3200 rpm for 15 minutes and separation of the plasma from the cells. All aliquots were stored at -20 °C until transfer under Arizona Department of Transportation guidelines to the University of Arizona for storage at -80 °C for subsequent processing by the University of Arizona Genetics Core. The CPT was processed according to the product guidelines and the cell pellet was stored at -80 °C until processed.

Genomic DNA from the EDTA tubes and CPTs was isolated using the FlexiGene DNA Kit (Qiagen, Valencia, CA). Genomic DNA was extracted from 9 additional packed cell pellets from CPTs using the Qiagen DNeasy Blood and Tissue Kit. DNA quantity was assessed with the QuantiFluor dsDNA System (Promega, Madison, WI) on the Synergy HT plate reader (BioTek Instruments, Inc., Winooski, VT) and 96 of the highest yield samples were normalized to 250ng in 30uL. The samples then underwent bisulfite conversion using the Zymo EZ DNA Methylation Kit (Zymo Research Corp., Irvine, CA) with a genomic DNA input of 250ng. The recommended modification to the protocol using alternative incubation conditions for the Illumina assays was performed. Upon bisulfite conversion completion, samples were sent to the University of Utah DNA Sequencing and Genomics Core Facility (Salt Lake City, Utah) for Infinium HD Methylation using the Illumina MethylationEPIC kit (Illumina, Inc., San Diego, CA) scanning on the iScan instrument, and raw data export.

Raw intensity data were processed by Bioconductor package minfi (version 1.22.1) [27] which included normalization of data using Illumina’s reference factor-based normalization methods (preprocess Illumina) and Subset-quantile Within-Array Normalisation (SWAN) [28] for type I and II probe bias correction. All samples passed quality control. A detection p-value is returned for every genomic position in every sample, with small p-values indicating good quality probes. Probes with detection p-value > 0.05 in one or more samples, and probes with single-nucleotide polymorphisms (SNPs) inside their body or at the nucleotide extension were excluded, leaving 834,912 probes. DNA methylation levels (M-values) were determined by calculating the logarithm of the ratio of intensities between methylated (signal A) and unmethylated (signal B) alleles, log (A/B) [28, 29]. Potential batch effects were investigated using principal component analysis using M-values.

### Statistical analyses

#### Differential methylation analysis

Differentially methylated probes were detected using the *limma* package [30]. A linear model with Empirical Bayes estimator was adopted [31], with adjustment for age, ethnicity, and BMI. Probes were considered to be differentially methylated if the resulting adjusted p-value was <0.05. The Bonferroni correction method was used to adjust the p-values and ensure that the familywise error rate was less than 0.05 [32]. The *DMRcate* package was used to identify differentially methylated regions (DMR) based on tunable kernel smoothing of the differential methylation signal, adopting the default setting [33]. *DMRcate* uses limma-derived statistics for calculation of individual CpG site methylation differences and it can assess all 850K probes as candidates for DMR constituents. The corresponding gene list was derived from the gene annotations associated with the probes. Because our DNA samples were derived from blood, we estimated white blood cell type composition for every individual using the Houseman method [34]. We corrected the analysis by including the estimated cell type composition as covariates in the linear model. Only results that were significant first without and then also with adjustment for cell type composition were reported as it has been shown that when cell composition and age are confounded, adjustment of cell-type composition can lead to false positives [35].

#### Genome-wide methylation prediction

Genome-wide methylation prediction was performed with the *glmnet* package using elastic-net penalization [36]. Years of service information was collected for both incumbent and new recruit firefighters. Since the newly recruited firefighters’ years of service measures were zero, we carried out a two-stage prediction model to incorporate this excess of zeros in the distribution of years of service. In the first stage we used genome wide methylation profile, age, BMI, and ethnicity to predict job status, i.e., recruit vs incumbent firefighter, which is equivalent to exposed to fire or not. In the second stage, for firefighters predicted to be incumbents, we then predicted their years of service. We employed a 10-fold cross validation strategy to repeatedly perform trainings on 90% of our sample set while holding out 10% of the samples for a test set. This procedure was repeated 10 times on unique subgroups of the entire data set.

#### Pathway analysis

We performed pathway analysis for the top probes differentially methylated between new recruits and incumbent firefighters using a p value of < 10^−4^ selected based on the published literature [37], and an empirically selected 1.5-fold change between the two groups. These probes were annotated to genes according to the closest transcription start site (TSS) [38]. The gene list was uploaded to QIAGEN Ingenuity Pathway Analysis (IPA, QIAGEN Redwood City) for assessing overrepresentation relative to all human gene functions [39]. The *Pathway Build* and *Relationship Summary* tools in IPA were used to build the gene regulatory networks, including expression regulation, protein-protein/DNA interaction, activation and inhibition. Genes were ranked by their connectivity in the regulatory networks, and genes with the top 10% connectivity were chosen as hubs. Hub genes play important roles in gene regulation due to their multiple interactions with other genes [40]. Two analyses were then performed to reveal the related canonical pathways and human diseases. First, using the *Canonical Pathways* tool, we identified canonical signaling (or metabolic) pathways with associated input genes and ranked the pathways by the number of gene members. Pathways that included more than five gene members were defined as top canonical pathways in this regulatory network. Second, using the IPA scientific literature-based *Diseases and Functions overlay* tool we annotated the genes enriched within human diseases and biological functions. The software is backed by highly structured, detail-rich biological and chemical findings derived from top journals and reviewed using full text and is also supported by third-party information, including but not limited to GO, TarBase, ClinicalTrials.gov, and BIND. It retrieves a wealth of experimental evidence for genes and explores the association with diseases or phenotypes by leveraging the depth of the Ingenuity Ontology and the Human Phenotype Ontology. With the IPA application, the significance of each enriched disease module is calculated as follows: (1) the number of input genes mapped to a given disease module in the IPA literature database, denoted by m; (2) the number of genes included in the disease module, denoted by M; (3) the total number of input genes mapped to the IPA’s literature database, denoted by n; and (4) the total number of known genes included in the IPA’s literature database denoted by N. The significance of gene enrichment in the disease module is then calculated using a one-tailed Fisher’s exact test [41]. Genes with no regulatory relationship with any other genes were excluded from analysis.

As IPA does not take the direction of the effects into consideration, directionality of methylation alteration was not assessed separately. Instead all alterations (whether gain of methylation or loss of methylation) were included in the analysis. The rational for this approach is that the methylation signature and associated alterations are a reflection of a cell’s transcriptional activity. Thus, regardless of direction, all methylation states in our samples of interest in theory contribute to the activity of cellular pathways.

## Results

### Subjects

**Fig 1:**
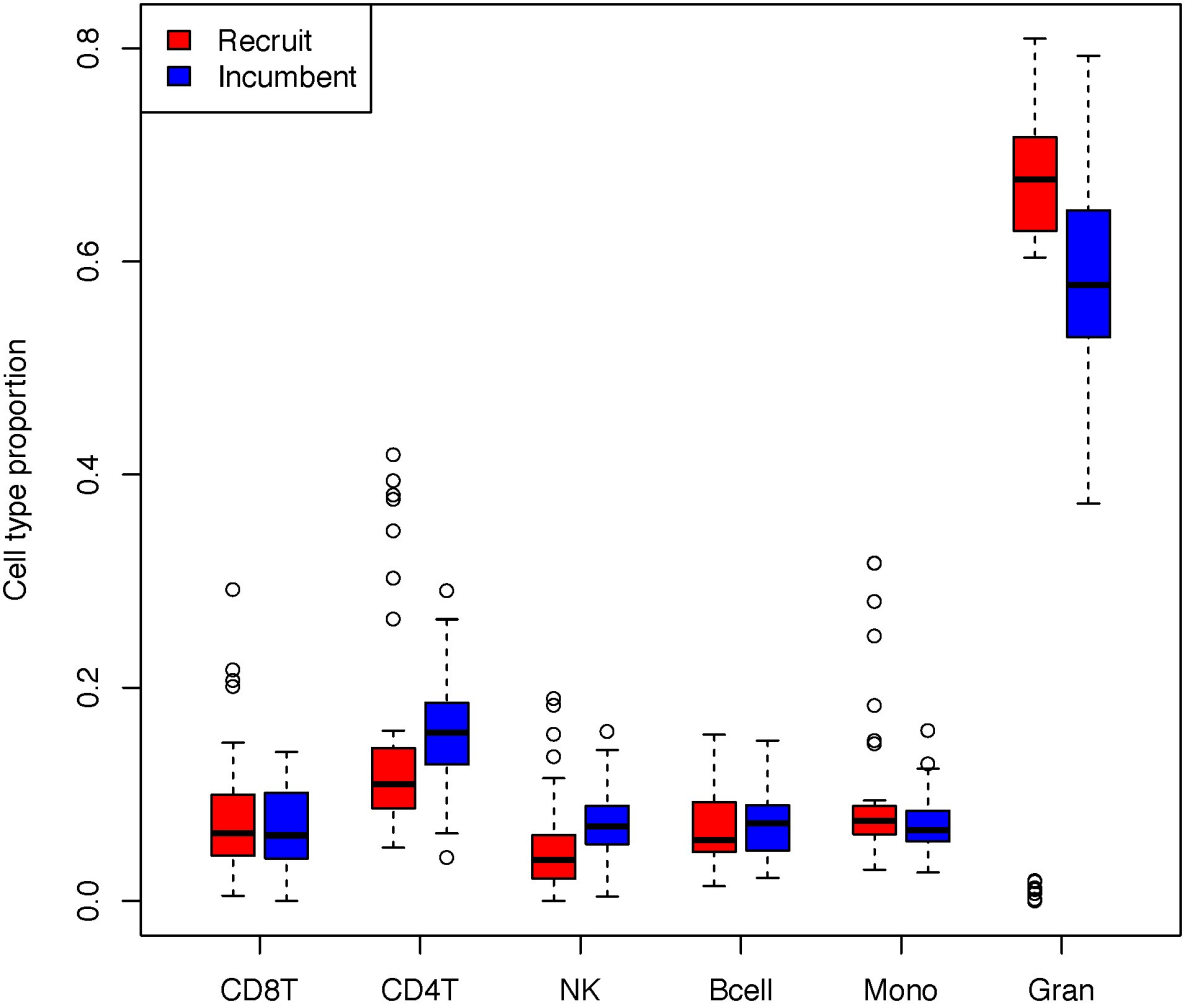
Cell type proportion among new recruit and incumbent firefighters.

All subjects were white, and a similar percentage of incumbent and new recruit firefighters were of Hispanic ethnicity (Table 1). The subjects’ mean age in years was significantly higher in incumbents (40.6 ± 7.7) than in recruits (28.9 ± 6.3) (p<0.001). The incumbent firefighters and recruits had similar distribution of BMI. For incumbents, the mean number of years serving as a firefighter was 14.0 ± 7.2 years, and number of years of service was significantly correlated with age (Pearson’s r = 0.804, p<0.0001). Distribution of cell type composition across job status is shown in Fig 1. There were no significant differences comparing the incumbent and new recruit firefighters.

**Table 1:** General characteristics of subjects.

Variable	Recruits (n = 41)	Incumbents (n = 45)	P-value
**Age (years)**			
≤ 29	23 (56.1%)	3 (6.67%)	<0.0001
30–39	14 (34.1%)	15 (33.3%)	
≥ 40	4 (9.76%)	27 (60.0%)	
Mean (SD)	28.9 (6.3)	40.6 (7.7)	<0.0001
**Body Mass Index (kg/m**^**2**^**)**			
Normal (18.5–24.9)	13 (31.7%)	9 (20.0%)	0.39
Overweight (25.0–29.9)	22 (53.7%)	26 (57.8%)	
Obese (≥ 30)	6 (14.6%)	10 (22.2%)	
**Race/Ethnicity**			
White, Hispanic	6 (14.6%)	6 (13.3%)	1.0
White, Non-Hispanic	35 (85.4%)	39 (86.7%)	
**Years of Service**			
Mean (SD)	0.85 (1.5)	14.0 (7.2)	< 0.0001
Missing	0	1 (2.2%)	

### Differential methylation analysis

Comparing incumbents to recruits and adjusting for multiple comparisons, age, BMI, and ethnicity, as well as cell type composition, four CpGs (cg00287370, cg05236728, cg12253469 and cg24034992) demonstrated statistically significant differential methylation exceeding 1.5-fold (Table 2). These four CpGs included one that was hypermethylated and three that were hypomethylated in incumbent firefighters compared to new recruits. Two of the three hypomethylated CpGs were annotated to promoter regions. One additional CpG (cg07897354) demonstrated significantly reduced methylation in incumbents as compared with recruits when adjusting for multiple comparisons, age and ethnicity, but lost significance when BMI was added to the model. In order to further assess the effects of age on differential methylation between incumbent and new recruit firefighters, we also investigated whether any of the CpG sites in Table 2 varied significantly by age group (<40 years old vs > 40 years old). None of these sites was significantly associated with age after correction for multiple comparisons (data not shown). In a separate analysis, 41 differentially methylated regions were identified, of which seven were still significant after adjustment for cell type compositions (Table 3).

**Table 2:** Differentially methylated positions.

CpG	Recruits^a^	Incumbent^a^	FC^b^	95% CI		Chr	UCSC RefGene Name	CpG Site Location	Regulatory Feature Group
				Lower	Upper				
cg12253469	98.7% (0.4%)	99.1% (0.3%)	2.40	1.81	3.20	22	MPST	Gene Body	
cg00287370	5.5% (0.9%)	3.7% (0.8%)	0.49	0.40	0.60	1			Promoter Associated
cg24034992	8.4% (1.3%)	5.2% (1.7%)	0.43	0.34	0.55	X	YIPF6	Gene Body	Promoter Associated Cell type specific
cg05236728	3.1% (0.9%)	2.0% (0.8%)	0.40	0.34	0.55	12	PCED1B	Gene Body; 5’UTR	
cg07897354^c^	4.4% (1.2%)	2.7% (0.9%)	0.44	0.34	0.58	18	SPIRE1		Promoter Associated

^**a**^Group mean (SD) of % methylation (Beta values).

^b^Fold changes (FC) of M values of CpG sites in incumbents compared to recruits with adjustment for age, ethnicity, and body mass index (BMI).

^c^Fold changes shown with adjustment for age and ethnicity; statistical significance lost when also adjusting for BMI.

**Table 3:** Differentially methylated regions.

Coordinate	Number of CpGs within region	Mean Beta FC within region^a^
chr19:37825009–37826008	12	0.07292553
chr19:52390810–52392100	15	0.05981331
chr12:47219626–47220197	13	0.08163149
chr19:12305392–12306303	10	0.03812897
chr15:29562049–29562633	10	-0.0089748
chrX:67719027–67719066	2	-0.0178829
chr14:64108940–64109325	5	-0.0089722

^a^Fold change comparing incumbents to new recruits after adjustment for age, body mass index (BMI), ethnicity and cell type composition.

### Genome-wide methylation prediction

**Fig 2:**
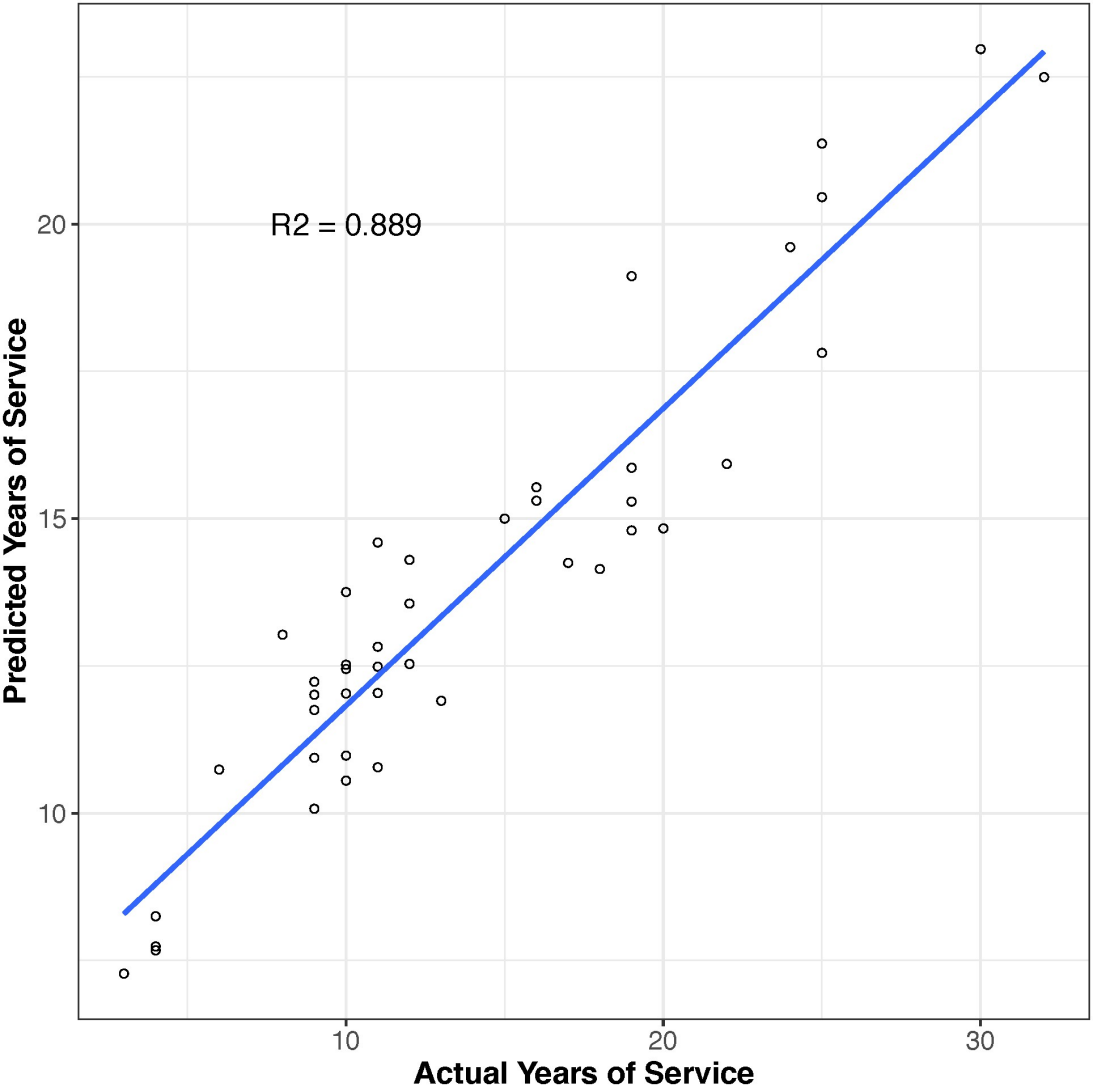
Predictive model for ‘years of service’ based on CpG level DNA methylation signals (n = 91).

Using a 10-fold cross validation procedure, we applied machine learning algorithms to determine which CpGs had variable methylation associated with firefighters’ service status, i.e., new recruit or incumbent, and the years of service each individual had performed. In each stage of cross validation, the CpGs that were determined by the training to be predictive were noted. A total of 91 CpGs were selected at least once during the 10 rounds of training associated with firefighters’ years of service. However, only 11 CpGs (cg09544149, cg24034992, cg22280238, cg00287370, cg02932780, cg13753209, cg15304928, cg07897354, cg22433210, cg20821958, and cg03177084) were selected in more than half of the trainings. The best-performing model was chosen based on the lowest misclassification rate in the first stage and the lowest mean squared error of years of service in the second stage in the test set. This model was then applied to the entire data set and predictions were compared to the actual years of service (Fig 2). The resulting misclassification rate between predicted incumbents and actual incumbent firefighters was 2% in the first stage and within incumbent firefighters the correlation of predicted and actual years of service was robust with an R^2^ of 0.889. We also evaluated whether including DNA methylation could increase predictive power compared to only using the covariates age, BMI, and ethnicity. By adding methylation levels to the prediction model, r^2^ increased from 0.533 to 0.889 and the misclassification rate was reduced from 8% to 2% (data not shown).

### Pathway analysis

**Fig 3:**
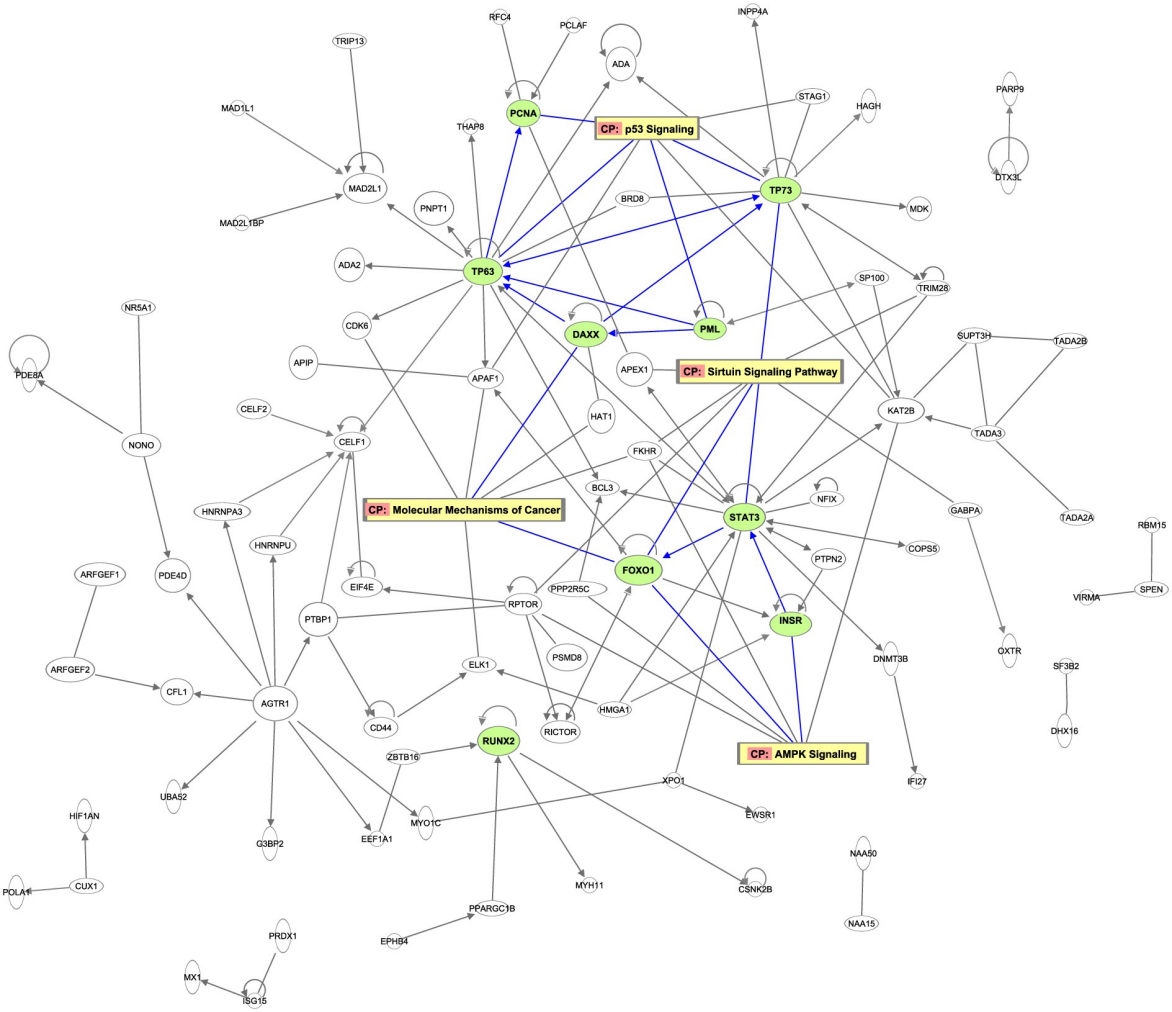
The gene regulatory network and pathways of enriched differential probes between new recruit and incumbent firefighters. Hub genes are highlighted in green. Top background and canonical pathways are highlighted in yellow. Connections between hub genes and top pathways are marked with blue lines.

Five hundred and twelve CpG sites demonstrated differential methylation with a *p*-value < 10^−4^ and at least 1.5-fold differences between incumbent firefighters and new recruits. They were annotated to 443 unique genes which were used to build a gene regulatory network (Fig 3). There were 93 genes that had at least one connection with other genes in the regulatory network. All hub genes had at least 20 connected relationships. They included *STAT3*, *TP63*, *TP73*, *FOXO1*, *PML*, *DAXX*, *RUNX2*, *INSR*, and *PCNA*. Top pathways with more than 5 gene members annotated from differentially methylated probes included the Sirtuin signaling pathway (3 hubs of 8 gene members: *FOXO1*, *STAT3* and *TP73*), molecular mechanisms of cancer (2 hubs of 7 gene members: *DAXX* and *FOXO1*), p53 signaling (4 hubs of 7 gene members: *PCNA*, *PML*, *TP63* and *TP73*), and 5' AMP-activated protein kinase (AMPK) signaling (2 hubs of 6 gene members: *FOXO1* and *INSR*). Enriched diseases (disease annotation) included abdominal cancer (9 hubs of 88 genes), colon tumor (8 hubs of 44 genes), skin cancer (6 hubs of 51 genes), and lung tumor/cancer (5 hubs of 49 genes), all with *p*-values <10^−6^ in IPA (Table 4). To address the effect of using a different fold change criteria, we performed a sensitivity analysis by using the same p-value < 10^−4^ but with a two-fold change limit (data not shown). The sensitivity analysis identified 293 CpG sites annotated to 282 unique genes (reduced from 512 CpG sites annotated to 443 genes in the primary analysis). Among them, 67 genes had at least one connection with other genes in the regulatory network based on IPA databases. Using the same hub gene criterion as in the primary analysis (i.e., with >20 connections with other genes in the regulatory network), six hub genes were identified, including *STAT3*, *PML*, *RUNX2*, *DAXX*, *PCNA*, and *INSR*. All of them were also reported in the primary analysis. The Sirtuin signaling pathway remained the top pathway with 5 annotated gene members, and the molecular mechanisms of cancer, p53 signaling, and AMPK signaling pathways all had at least 3 gene members.

**Table 4:** Disease annotation, number of related genes, and the corresponding hubs.

Disease annotation	*p*-value	# of genes	Hub genes
Abdominal cancer	5.1e-18	88	*STAT3*, *TP63*, *TP73*, *FOXO1*, PML, *DAXX*, *RUNX2*, *INSR*, *PCNA*
Abdominal neoplasm	2.2e-19		
Abdominal carcinoma	1.1e-11		
Adenocarcinoma	5.4e-16		
Colon tumor	5.9e-09	44	*STAT3*, *TP63*, *TP73*, *FOXO1*, *DAXX*, *RUNX2*, *INSR*, *PCNA*
Skin cancer	2.9e-07	51	*STAT3*, *TP63*, *PML*, *DAXX*, *RUNX2*, *INSR*
Lung tumor	6.6e-07	49	*INSR*, *PCNA*, *STAT3*, *TP63*, *TP73*
Lung cancer	1.0e-06		

## Discussion

The results of this study support our hypothesis that, compared to new recruits, incumbent firefighters would show differential DNA methylation associated with cancer pathways. This adds to the currently sparse body of literature describing the epigenetic effects among firefighters, a population occupationally exposed to known carcinogens with documented increased cancer risk [2].

Our differential methylation analysis identified five CpGs assigned to both promoter and non-promoter regions. Promoter hypermethylation frequently leads to silencing of tumor-suppressor or DNA repair genes in cancers while hypomethylation of CpGs often results in overexpression of genes [22, 42–44]. However, recent investigations of broader methylation patterns suggest that non-promoter (intragenic) methylation may also affect transcription regulation and efficiency; while CpG hypermethylation in non-promoter regions does not impede transcription (as it does in promoter regions), it has been correlated with increased or ectopic gene expression [45–48].

Four of the five differentially expressed CpG sites are located in genes with known functions and reported associations with cancer and metastatic potential. However, all five differentially expressed CpGs identified in this study represent novel epigenetic markers that have not previously been reported in the limited body of literature describing differential DNA methylation in firefighters or those with similar occupational exposures. One CpG with decreased methylation among incumbent firefighters was located on the *YIPF6* gene, annotated to the promoter region. *YIPF6* has been associated with prostate cancer, and amplification and overexpression of *YIPF6* protein has been posited to indirectly stimulate tumor progression [49, 50]. Another CpG with decreased methylation in incumbent firefighters is located in the gene body of *PCED1B*. This gene encodes a protein that belongs to the GDSL/SGNH-like acyl-esterase family, hydrolases thought to function in modification of biopolymers on the cell surface. High expression of this gene has significant associations with renal (unfavorable) and urothelial cancer (favorable) patient survival based on Cancer Genome Atlas (TCGA) data (https://www.proteinatlas.org/ENSG00000179715-PCED1B/pathology). One CpG with decreased methylation in incumbent firefighters, that was statistically significant until additionally adjusted for BMI (Table 2), is located in the promoter region of *SPIRE1*. The dysregulated expression of the protein encoded by this gene, SPIRE1, has been associated with cellular potential for extracellular matrix degradation, which may impact the invasive and metastatic behavior of cancer cells [51]. The hypermethylated CpG identified in our analysis was located on the *MPST* gene body. The *MPST* encoded protein is associated with cysteine degradation, cyanide detoxification and likely other metabolic processes, given observed *MPST* deficiency in individuals with the heritable disorder, mercaptolactate-cysteine disulfiduria [52]. As part of its cysteine degradation pathway, *MPST* produces enzymes involved in formation of sulfane sulfur containing compounds. Sulfur metabolism dysregulation in cancer cells and anti-cancer effects *in vivo* of sulfane sulfur precursors suggest that proliferation of malignant cells may be related to a deficiency of sulfane sulfur and the uncontrolled operation of a set of enzymes normally inactivated by sulfane sulfur [53].

Of the seven differentially methylated regions that remained significant after correction for cell type composition, three were located on genes (*SYNE2*, *AR*, and *PCED1B*) with known functions and disease associations. *SYNE2* encodes a protein involved in maintaining the structural integrity of the nucleus. *AR*, the androgen receptor gene, encodes a member of the steroid hormone nuclear receptor family that regulates gene expression. AR signaling is reported to be involved in prostate, bladder, liver, kidney and lung tumorigenesis and metastasis [54, 55]. Differential methylation patterns of *AR* are also associated with prostate cancer, non-Hodgkin's lymphoma, and ovarian cancer [24, 56–58]. Variants of *SYNE2* have been associated with p21 expression and reduced overall survival in hepatitis B-related hepatocellular carcinoma [59]. p21 is a cell cycle regulator reported to downregulate TP53, a tumor suppressor [60, 61]. Known functions of *PCED1B*, which also contained a differentially methylated CpG as shown in Table 2, were previously discussed above. Information for the four remaining regions were sparse. One region was located on the protein coding gene *FAM189A1*, which is reported to have tissue-specific expression in brain and colon, but no known disease associations [62]. No information about function or disease associations was found for *ZNF528-AS1*. One region was located on or near uncharacterized genes (*AC016582*.*2* and *CTD-2554C21*.*2*) and for the region containing chr19:12,305,392–12,306,303 no further information was available.

Because DNA methylation signatures are tightly correlated to transcriptional activity throughout the genome, they provide a powerful platform for prediction of complex traits or diseases [63–65]. Our machine learning analyses were used to predict whether or not an individual was an incumbent firefighter (and had thus had a certain anticipated level of environmental exposures) and how long that individual had been in the service. Five of the 11 CpGs identified in our best-performing predictive model, cg24034992, cg02932780, cg15304928, cg07897354, and cg03177084, were located on or near genes *YIPF6*, *VARS*, *TMEM9*, *SPIRE1*, and *PSME3*, respectively. *YIPF6*, *TMEM9*, and *PSME3* have been associated with cancer [49, 50, 66–69] and *SPIRE1* reportedly contributes to metastatic potential [51]. *VARS* encodes a multi-domain protein that catalyzes the aminoacylation of tRNA and has been associated with neurodevelopmental disorders [70]. No information was available for the remaining 6 CpGs.

The top identified canonical pathways with differentiated methylated genes included many associated with cancer. The sirtuins, which regulate a large number of cellular pathways and protect the age-associated diseases, regulate processes in cancer cells such as DNA repair and cancer metabolism [71, 72]. More than half of all cancers may involve p53-inactivating mutations, and downstream p53 signals result in cell cycle arrest, apoptosis or senescence [72–74]. AMPK, a highly conserved kinase through evolution, regulates energy-consuming biosynthetic pathways, and activation of AMPK by pharmacological or other means might reduce cancer incidence [75, 76]. The *STAT3* gene, the top identified hub, is a component of essential chemical signaling pathways within cells and an ideal target for chemoprevention and cancer therapy [77, 78]. *STAT3* acetylation silences gene expression and enhances DNA methylation of key tumor-suppressor gene promoters, and inhibition of *STAT3* acetylation reverses aberrant CpG island methylation and leads to the reactivation of several tumor-suppressing gene promoters [79]. Overexpression of *STAT3* leads to continued growth of tumor cells and promotes other malignant properties such as tumor angiogenesis [80, 81]. Tumor proteins p63 and p73, encoded by the *TP63* (on p53 pathway) and *TP73* genes (on both p53 and Sirtuin pathway), provide a complex contribution to tumorigenesis as they regulate cell cycle and apoptosis after DNA damage. For example, *TP73* has been found to be transcriptionally silenced in lymphoblastic leukemias and lymphomas induced by CpG island methylation [82–84]. p63 genomic amplification may have an early role in lung tumorigenesis and may act as a biomarker for lung cancer progression [84]. *INSR*, has been used as a biomarker for prognosis of non-small cell lung cancer and an INSR protein inhibitor, Zykadia, has been authorized by U.S. Food and Drug Administration (FDA) [85] and European Medicines Agency [86] as a treatment of advanced ALK-positive non-small cell lung cancer [87].

Increased risk of many of the enriched diseases identified in our pathway analysis (abdominal cancer, adenocarcinoma, colon tumor, skin cancer, lung cancer) have been previously reported among firefighters. A study examining firefighters from Nordic countries reported excess risk of adenocarcinomas among firefighters aged 70 years and older [6]. In a pooled cohort of US firefighters, excess cancer mortality and incidence were reported for digestive and respiratory sites, including colorectal, mesothelioma and lung cancers [2]. A higher risk of colorectal cancer was also observed in a 2006 meta-analysis of 32 studies on firefighters [5]. Several studies have also reported higher prevalence and risk of non-melanoma and melanoma skin cancer among firefighters [5–7, 88].

Firefighters are exposed to elevated concentrations of multiple products of combustion and other toxic substances, including PAHs, benzene, and PFAS, many of which are carcinogenic, genotoxic or mutagenic [11, 13, 89–92]. Studies among other highly exposed populations have reported associations between PAH exposure and global or gene promoter-specific DNA methylation changes, suggesting that these epigenetic changes may reflect a history of exposure to PAHs [93, 94]. Firefighters also generally work in shifts, typically 24 hours, and shiftwork that disrupts circadian rhythms has been concluded to be “probably carcinogenic” [16]. Studies in non-firefighter populations have also found that long-term shiftwork is associated with differential DNA methylation and whole-genome methylation [95, 96] and there is increasing evidence that long-term shiftwork may increase the risk of breast cancer via epigenetic mechanisms [96–98]. Additional studies are needed, ideally prospective cohort studies with a larger number of firefighters, to help validate the specific CpG sites identified in the current study and to determine which exposures are associated with altered methylation at those sites.

Prior published studies on the relationship between firefighter occupational exposures and epigenetic changes are scarce. We could find only one other study focused on differential DNA methylation among firefighters. This study assessed promoter methylation in four *a priori* genes comparing firefighters to non-firefighting controls and reported significant decreased methylation for one of the four genes, *DUSP22*, as well as a correlation between duration of firefighting service and decreased methylation [20]. However, in our analysis we were unable to detect significant differential DNA methylation at the *DUSP22* promoter region. Additionally, the previous study demonstrated that the decreased *DUSP22* promoter methylation was inducible in cultured human cells by low-dose exposure of benzo[a]pyrene, a highly carcinogenic PAH [20]. In our previous analysis based on many of the same Arizona firefighters evaluated in the current study, we identified nine miRNA markers differentially expressed in incumbent firefighters compared to new recruits [19]. Notably, the six miRNAs with reduced expression in incumbent firefighters have reported tumor suppressor activities while two of the three miRNAs with increased expression are reported to participate in cancer promoting activities, consistent with the hypothesis that firefighters are at increased cancer risk.

The results of our study provide potential mechanisms linking firefighter exposures and the excess risks of specific cancer types identified in epidemiologic studies of cancer in the fire service [1–8]. Given the long latency between exposures and the development of cancer, ranging from less than 5 years to greater than 30 depending on the type of cancer, DNA methylation biomarkers have the potential to be used to both identify the cumulative effect of exposures and to identify firefighters at increased risk of disease susceptibility. In addition to its use in helping to predict future disease, DNA methylation could potentially be used to assist in determining cancer diagnosis and prognosis, as has been demonstrated in groups other than firefighters [99–101]. For example, the methylation signature identified can be used as an “epigenetic clock” of firefighting. If the magnitude and/or length of exposures is both predictive of cancer risk and detectable in methylation signatures, it is likely that prediction of future cancer risk may eventually be attainable. If this is true, it is possible that preventative efforts and close monitoring can be put in place for firefighters at particularly high risk. Identification of epigenetic markers both associated with exposures in firefighters and diseases also have the potential to assist in determining occupational causation in workers’ compensation cases.

Limitations of the current study include a relatively small sample size, a cross-sectional design, and inclusion of firefighters from a single geographic region. There was also a significant age difference between the incumbent and new recruit firefighters, although we adjusted for age in our analyses. To further ensure that age differences were not driving the differential methylation identified in our study, we assessed the CpGs known to be altered with age and compared them to the CpGs identified in our study. We did not identify any overlap in these significant regions (data not shown). Future longitudinal studies of a larger number of firefighters across geographic regions are needed to determine the extent to which the findings can be generalized to other firefighter populations, ideally with an external comparison group of similar age to the incumbent firefighters. Information on lifestyle exposures was limited to smoking; no information on diet was available, and occupational history was limited to years as a firefighter. It will also be important in future studies to determine the association among this broader group of exposures with the identified DNA methylation markers, as well as to determine whether the markers are predictive of disease outcomes in firefighters.

## Conclusions

In conclusion, DNA methylation varied among male non-smoking incumbent firefighters and new recruits after adjusting for age, BMI and ethnicity. Furthermore, DNA methylation markers were also able to predict with high accuracy the number of years worked as a firefighter. Based on pathway analysis, many of the DNA methylation markers were associated with cancer, supporting the potential for these changes to help explain the mechanism for increased cancer risk among firefighters.
